# Investigating the temporal dynamics of electroencephalogram (EEG) microstates using recurrent neural networks

**DOI:** 10.1002/hbm.24949

**Published:** 2020-02-24

**Authors:** Apoorva Sikka, Hamidreza Jamalabadi, Marina Krylova, Sarah Alizadeh, Johan N. van der Meer, Lena Danyeli, Matthias Deliano, Petya Vicheva, Tim Hahn, Thomas Koenig, Deepti R. Bathula, Martin Walter

**Affiliations:** ^1^ Department of Computer Science and Engineering Indian Institute of Technology Ropar, Rupnagar Punjab India; ^2^ Department of Psychiatry and Psychotherapy, Division for Translational Psychiatry University of Tübingen Tübingen Germany; ^3^ QIMR Berghofer Medical Research Institute Brisbane Australia; ^4^ Clinical Affective Neuroimaging Laboratory Magdeburg Germany; ^5^ Leibniz Institute for Neurobiology Magdeburg Germany; ^6^ Department of Psychiatry Otto von Guericke University of Magdeburg Magdeburg Germany; ^7^ Institute of Translational Psychiatry, University of Muenster Muenster Germany; ^8^ Translational Research Center University Hospital of Psychiatry, University of Bern Bern Switzerland; ^9^ Max Planck Institute for biological cybernetics Tübingen Germany; ^10^ Department of Psychiatry and Psychotherapy Jena University Hospital Jena Germany

**Keywords:** EEG, microstates, recurrent neural networks, stress

## Abstract

Electroencephalogram (EEG) microstates that represent quasi‐stable, global neuronal activity are considered as the building blocks of brain dynamics. Therefore, the analysis of microstate sequences is a promising approach to understand fast brain dynamics that underlie various mental processes. Recent studies suggest that EEG microstate sequences are non‐Markovian and nonstationary, highlighting the importance of the sequential flow of information between different brain states. These findings inspired us to model these sequences using Recurrent Neural Networks (RNNs) consisting of long‐short‐term‐memory (LSTM) units to capture the complex temporal dependencies. Using an LSTM‐based auto encoder framework and different encoding schemes, we modeled the microstate sequences at multiple time scales (200–2,000 ms) aiming to capture stably recurring microstate patterns within and across subjects. We show that RNNs can learn underlying microstate patterns with high accuracy and that the microstate trajectories are subject invariant at shorter time scales (≤400 ms) and reproducible across sessions. Significant drop in the reconstruction accuracy was observed for longer sequence lengths of 2,000 ms. These findings indirectly corroborate earlier studies which indicated that EEG microstate sequences exhibit long‐range dependencies with finite memory content. Furthermore, we find that the latent representations learned by the RNNs are sensitive to external stimulation such as stress while the conventional univariate microstate measures (e.g., occurrence, mean duration, etc.) fail to capture such changes in brain dynamics. While RNNs cannot be configured to identify the specific discriminating patterns, they have the potential for learning the underlying temporal dynamics and are sensitive to sequence aberrations characterized by changes in metal processes. Empowered with the macroscopic understanding of the temporal dynamics that extends beyond short‐term interactions, RNNs offer a reliable alternative for exploring system level brain dynamics using EEG microstate sequences.

## 1 INTRODUCTION

Four quasi‐stable states explain consistently around 80% of total topographic variance in spontaneous electroencephalography (EEG). These states are referred to as microstates and have been suggested to be the “building blocks of brain functions” (Khanna, Pascual‐Leone, Michel, & Farzan, 2015; Koenig et al., 2002; Lehmann & Michel, 2011; Michel & Koenig, 2017). Recent studies show that changes in the properties of microstates (e.g., mean duration) are associated with neuro‐psychiatric disorders (Michel & Koenig, 2017), for example, schizophrenia (Andreou et al., 2014; Lehmann et al., 2005; Rieger, Diaz Hernandez, Baenninger, & Koenig, 2016), depression (Damborská et al., 2019; Strik, Dierks, Becker, & Lehmann, 1995), epilepsy (Pittau, Baldini, Tomescu, Michel, & Seeck, 2018), as well as stages of development (Koenig et al., 2002). The continuous time course of microstate appearances exhibits long range dependencies over at least six dyadic scales (Van de Ville, Britz, & Michel, 2010) and is interrelated with some of the well‐known blood oxygen‐level dependent (BOLD) resting state networks (Britz, Van De Ville, & Michel, 2010; Rajkumar et al., 2018; Van de Ville et al., 2010), effectively associating them, among others, with visual, auditory, and attention processes (Milz et al., 2016; Seitzman et al., 2017). Therefore, studying the trajectory of microstates which represents the whole brain dynamics, and seems to be governed by distinct but interconnected processes, is a promising venue to investigate the brain dynamics at the system level.

However, EEG trajectories are rarely investigated by proper mathematical tools capable of modeling the dynamics in a sequence‐preserving way. More recently, modeling techniques based on hidden Markov models (Gschwind, Michel, & Van De Ville, 2015), random walk (von Wegner, Tagliazucchi, Brodbeck, & Laufs, 2016), and stochastic process (von Wegner, Tagliazucchi, & Laufs, 2017) are gaining momentum to investigate the transition properties of microstates. Such approaches are nevertheless limited in terms of their dynamical richness (Gschwind et al., 2015). A fundamental issue with the transition matrix approach is the combinatorial increase of the number of possibilities when the length of sequences is extended, which leads to a sharp decrease in the reliability of models. Consequently, adequate modeling of microstates sequences needs to reach beyond simply the sequence of labels and should consider temporal relations within and between states (Gschwind et al., 2015).

In this article, instead of trying to explicitly model the temporal dynamics of EEG microstates, we ask if there are any temporal patterns in the sequence of EEG microstates that can be reliably and reproducibly detected. This question is best addressed using recurrent neural networks (RNNs) that are known to be a rich and flexible methodology to learn complex temporal dependencies without making any assumption on the temporal characteristics of the signal. Deep neural networks with a recurrent structure (i.e., RNNs) have been used successfully to model various temporal sequences (Cho et al., 2014; Sutskever, Vinyals, & Le, 2014). Unlike conventional feed‐forward neural networks that consider all samples to be independent, RNNs have loops with a chain‐like structure that dynamically engage information learned from the past to be used for future samples and therefore, have been employed to understand videos and temporal data with promising results (Cho et al., 2014; Venugopalan et al., 2014). Specifically, an RNN consisting of long‐short‐term‐memory (LSTM) cells has been proven to be successful in modeling temporal dependencies in sequential data (Hochreiter & Schmidhuber, 1997; Sak, Senior, & Beaufays, 2014). With short‐term memory that can last for a specific period of time, LSTM is well‐suited to process, analyze, and predict sequential data with unknown time lags and durations. Therefore, LSTM networks seem to be suitable for modeling patterns in EEG microstates that are quasi‐stable and transient. While sequence‐to‐sequence autoencoders (AE) have been employed successfully in several tasks such as machine translation and video‐to‐text (Cho et al., 2014; Venugopalan et al., 2014), to the best of our knowledge, there is no previous work on extracting representations from EEG microstate sequences using this method.

The proposed model aims to learn the underlying patterns that exist in EEG microstate sequences where these states exhibit quasi‐stability. Use of RNNs alleviates the need to predefine features which aids in learning potential nonlinear microstate dynamics directly from the data. LSTM architecture goes beyond the step‐by‐step short‐term interactions (modeled by conventional methods like Markov Chains) to capture potentially existing long‐range dependencies. Specifically, we explore the temporal dynamics of the microstate sequences using an LSTM‐based AE neural network, that is, trained to reconstruct its input to the output, by first compressing the input into a latent‐space representation and then using this representation to reconstruct the output. Effectiveness of the proposed model in representing the complex dynamic structure is demonstrated through accurate reconstruction of microstate sequences. Further, we try to study the patterns learned by the RNNs by visualizing LSTM cells that react to specific patterns in microstate trajectories to gain intuition into the internal learning mechanisms and the patterns of EEG microstates that govern the temporal structure of microstates. We show that the model reacts to the changes in microstate sequences after stress induction and that it has the ability to categorize stress‐induced condition from baseline microstate sequences which further demonstrates the potential of the learned representations for applications such as classification and cross‐modality estimations. Finally, as RNNs have the sequence prediction capability, we attempted to forecast future states in the microstate sequence by combining the historical sequence information with the learned internal representation. Relatively low prediction accuracies beyond a few milliseconds corroborates the nonstationary nature of the resting state microstate sequences due to irregular structure of microstate durations.

## 2 MATERIAL AND METHODS

### 2.1 Data acquisition

Here, we used two datasets to assess the temporal structure of EEG microstates. The first dataset is from a study which employs EEG data with simultaneous Functional magnetic resonance imaging (fMRI) where we have access to the resting state recorded pre and post stress condition which is of interest to study its effect on temporal properties of EEG microstates. Furthermore, possible shortcomings of EEG artifact correction recorded inside magnetic resonance imaging (MRI) scanner were then controlled using the second dataset which is recorded outside MRI scanner.

*Dataset 1A*: Data were obtained from simultaneous 3T EEG‐fMRI recordings of 12‐min eyes‐closed resting‐state of 34 healthy male volunteers (mean age 44.06 ± 9.96). EEG data were acquired using the BrainAmp MR system (Brain Products) with a 64‐channel Easycap augmented with six carbon‐wire‐loops (CWLs; van der Meer et al., 2016). One channel placed on the back was used for electrocardiogram (ECG) detection. FCz was used as reference electrode and CPz as ground electrode. The sampling rate was 5,000 Hz

*Dataset 1B*: Data were obtained from simultaneous 3T EEG‐fMRI recordings during a 12‐min eyes‐closed resting‐state following a psychosocial stress paradigm in 34 male subjects (mean age = 43.26 ± 10.19). EEG and fMRI data were acquired with the same procedure as in *Dataset 1A* with the only difference that resting‐state data was recorded after subjects underwent a psychosocial stress paradigm. The stress paradigm was an adapted version of the ScanSTRESS task as described by (Streit et al., 2014) where subjects were asked to perform two tasks containing mental rotation and arithmetic calculation. For both types of tasks, control blocks were without any social evaluative feedback, time pressure, or difficult questions and stress blocks were with feedback about the correctness and speed of the answers as well as more demanding questions. During the whole experiment, subjects were exposed continuously to a video feed of the reactions of two panel members, who were passive during the control blocks but reacted disapprovingly to the participant's performance during the stress condition. In total, there were four 40‐s blocks of each type (arithmetic control/stress and mental rotation control/stress) presented in two runs. After the first run (consisting two blocks of each condition), the experiment was interrupted for an extensive, negative, verbal feedback from the panel members stating that the performance was poor and more effort necessary or the data would not be usable

*Dataset 1* was recorded in 39 subjects who underwent baseline and post psychosocial stress recordings in the scanner twice, that is, on 2 days, where there was a period of 7–35 days between 2 days. The EEG acquisition was performed adjacent to a clinical trial (NCT02602275) where after the baseline measurements subjects took either placebo or an herbal medicinal product (Neurexan) in a counterbalanced order. Participants who received placebo on the first day took Neurexan on the second day and vice versa. This article only reports results analyzed for the days on which placebo was taken. The data from five subjects in each resting state were excluded from the current analysis because of the problems with recording and/or the low quality of their available EEG data.

*Dataset 2* EEG data were obtained in a shielded cabin from 15‐min eyes‐closed resting‐state in 11 healthy male volunteers (mean age = 24.42 ± 3.05). EEG data were acquired using a BrainAmp (Brain Products) with 64‐channel actiCap. Two bipolar electrodes placed on the right and the left arm were used for ECG detection and two further bipolar electrodes with galvanic skin response‐module input were used for skin conductance measurements. FCz was used as reference electrode and AFz as ground electrode. The electrode PO10 was used for eye movement detection and was excluded from the further analysis. The sampling rate was 2,500 Hz, but data were down‐sampled to 1,000 Hz.

### 2.2 Data preprocessing

Artifact rejection for EEG data was done in a semiautomatic process using custom MATLAB scripts. First, the raw EEG data was bandpass filtered between 0.3 and 200 Hz. Then, EEG was cleaned from MRI gradient artifacts by motion informed template subtraction technique (Moosmann et al., 2009). Then, the helium pump and ballisto‐cardiac artifacts were removed using the CWLs artifact correction technique (van der Meer et al., 2016). Finally, the data were segmented into 2 and 1 s trials, for *Datasets 1 and 2* respectively, and the trials containing muscle and head movement artifacts were removed from the dataset. Data segmentation was necessary to allow removal of the data containing muscle and head‐movement‐related artefacts. Because *Dataset 1* contains MRI artefact, we aligned EEG segmentation to the repetition time of the simultaneous MRI scanner. *Dataset 2* was recorded outside the scanner and therefore we were able to use a finer segmentation (here 1 s), that allows us to be more precise in the artefact removal and keep more data.

The channels that contained too many epochs with artifacts were also removed and interpolated using routines provided by EEGLAB (Delorme & Makeig, 2004). The latter step of the artifact rejection process also includes independent component analysis decomposition of the EEG data and removing the components that reflected eye movements, continuous muscle activity, and residual MRI‐artifacts. Note that since *Dataset 2* was recorded outside of the scanner, we only applied the non‐MRI part of the pipeline on this dataset.

### 2.3 Microstate extraction

**Figure 1:**
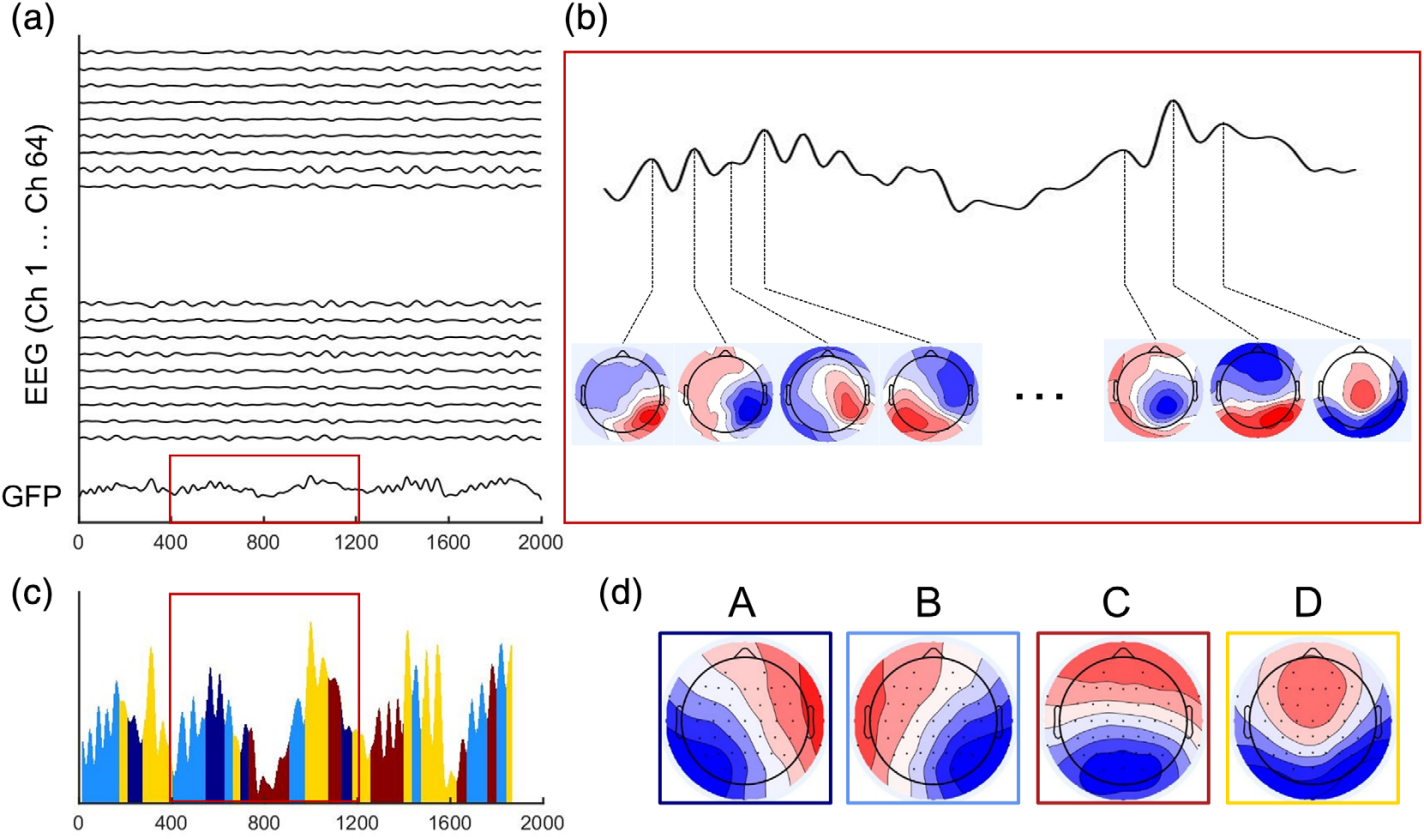
Schematic representation of microstate extraction. (a) The global field power (GFP) is calculated at each time point of the multichannel electroencephalogram (EEG) recording. (b) At peaks of the GFP curve, the potential recorded at each electrode of the multichannel signal is plotted onto a map of the channel array. (c) The head‐surface topographies of the four EEG microstate classes for *Dataset 1*. (d) The original maps at peaks of the GFP curve are assigned to a microstate Class A, B, C, or D based on the degree of correlation with the microstate maps

To extract EEG microstates, we used the EEGLAB plugin developed by Thomas Koenig (http://www.thomaskoenig.ch/index.php/software/). Artifact‐free EEG was band‐pass filtered between 1 and 40 Hz, down‐sampled to 250 Hz, and the peaks of the global field power (GFP) were determined after convolving the GFP time series with a Gaussian filter of 10 time‐points window length. We use GFP to assign the microstate labels as they are suggested to be the best representation of instantaneous EEG topographies (Koenig, Studer, Hubl, Melie, & Strik, 2005). All maps marked as GFP peaks were extracted and submitted to a modified k‐means clustering algorithm to deduce the four classes of map topographies that maximally explain the variance of the map topographies. These four classes of map topographies were then submitted to a full permutation procedure to compute mean classes across participants. Full permutation procedure is a permutation algorithm that is dedicated to maximize the common variance over the subjects. This is done in an iterative procedure by swapping individual microstate topographies for best fit of the prototype maps and updating the prototypes by calculation grand average over subjects (see Koenig et al., 1999 for further details). Using the mean microstate classes across subjects as templates, for all participants the EEG topographies at the local maxima (peaks) of the GFP were assigned to one of these four microstate classes based on maximal Pearson correlation (see Figure 1). Time points between GFP peaks were assigned to the microstate class of the temporally closest GFP peak. Successive maps assigned to the same class were recognized as belonging to one microstate. Finally, the temporal dynamics of microstates are conventionally quantified in terms of the average duration of microstates each time they occur (i.e., Duration), the number of times they occur in a second (i.e., Occurrence), and the proportion of time spent in each microstate (i.e., Contribution; Khanna et al., 2015).

### 2.4 Reconstruction of microstate sequences using RNN

**Figure 2:**
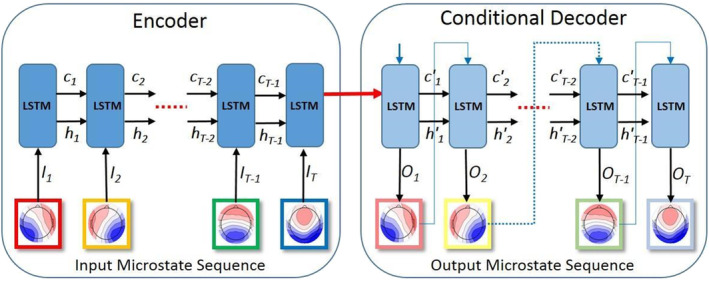
Encoder–Decoder architecture of the long‐short‐term memory (LSTM) network. The architecture employs two LSTM networks called the encoder and decoder. The encoder is a single layered recurrent neural networks (RNN) with *N*_*u*_ units of LSTM. At each time step, the hidden state of the encoder is updated based on the input microstate. Therefore, the final hidden state of the encoder RNN contains information about the whole input sequence. This final hidden state is used by the decoder RNN to recreate the original sequence by minimizing the reconstruction error. The decoder RNN is structurally similar to the encoder with same number of layers and LSTM units

We use a recurrent sequence‐to‐sequence AE framework (see Figure 2) to learn high‐level, compact representations of EEG microstate sequences. The AE is trained in an unsupervised setting to read the input sequence, encode it and finally decode it to recreate the sequence accurately. Unlike traditional neural networks, RNNs are designed to recognize patterns in sequential data.

The encoder processes the input microstate sequence, *I*_1_, *I*_2_, ⋯, *I*_*T*_ of length *T* and summarizes the observed temporal pattern in the form of latent representation (hidden state). The task of the decoder is the state‐by‐state reconstruction of the input microstate sequence. The reconstruction is based on the latent representation learned by the encoder RNN which is used to initialize the hidden states of the decoder RNN (see Figure 2).

In this work, we employ an architecture similar to the LSTM‐based AE that was first employed by Srivastava, Mansimov, & Salakhudinov (2015) to learn representations of spatiotemporal information in video sequences. As illustrated in Figure S1, each LSTM unit has a memory cell and a set of gates that control the flow of information. A chain of such LSTM units is organized into an Encoder‐Decoder architecture (see Figure 2) to learn the latent representation of microstate sequences that not only summarizes the high‐level patterns contained in the microstate sequences but also learns the temporal dependencies between subsequent states.

In particular, the LSTM‐based AE is fed with microstate sequences to encode temporal patterns that are stable across and within subjects. As EEG microstate is a categorical variable with labels *A* to *D*, one hot encoding is used to first convert it into numerical form. Here, each label is mapped to a binary vector with a single non‐zero entry (1,0,0,0), (0,1,0,0), (0,0,1,0), (0,0,0,1) such that the pairwise distances between all microstates are the same. At each step, the network receives a sequence of EEG microstates and generates an output sequence of the same size. EEG sequence is sliced into segments of size *S* that represents the length of the sequence in time points and input to the LSTM network has dimensions of *S* × 4 due to one hot encoding.

### 2.5 Intermediate representation

**Figure 3:**
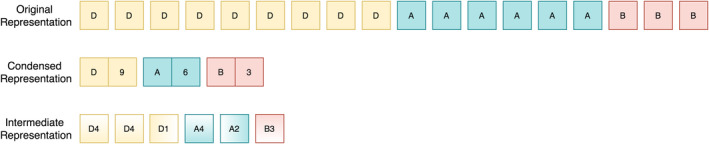
Different microstate representations that were used to encode long‐term dependencies. Original representation is the most intuitive encoding scheme and requires only four symbols. On the other extreme, condensed representation is sparse but also minimalistic. Intermediate representation provides a tradeoff between these two by assigning different symbols for microstate durations up to four time points and arranging them in decreasing order. With only fourfold increase in the number of symbols, intermediate representation strikes a balance between recurrence and sparsity

Technically, only four symbols are enough to represent each of the microstates where consecutive appearances of a microstate represent its local persistence in a sequence. However, duration of a microstate is very irregular and ranges from 0 to 300 ms. As persistence could be a dominating factor that overwhelms the training process and is also shown to be an important encoding feature of microstates (Khanna et al., 2015), alternative encodings were considered to include this information in the samples. The most compact representation that encodes the temporal information would be to encode each microstate along with its persistence as a unique symbol as shown in Figure 3. However, due to the heavy‐tailed distributions of EEG microstate durations (Gschwind et al., 2015), such condensed representation will lead to a large number of symbols in the new representation which will make it too sparse compared to number of instances and therefore, cannot reflect the distribution characteristics of the sequence correctly. For example, although microstate B occurs with duration of 100 ms very rarely, it still gets a unique symbol under condensed representation. To overcome this issue, we adapt an intermediate encoding scheme (as shown in Figure 3) that represents a compromise between the original and compact representations. Although many such intermediate representations are possible, we chose to encode microstates persisting up to four time points with different symbols (A1–A4, B1–B4, etc.) and string them in decreasing order (e.g., D9 is coded as D4 D4 D1). This representation offers a tradeoff between recurrence and sparsity as the number of symbols only increases from four to sixteen while sustaining the notion of perseverance. Importantly, this allows to dissect the possible effect of limited memory capacity of the RNNs from intrinsic temporal structure of EEG microstates should the reconstruction accuracy differ for various microstate sequence durations.

### 2.6 Generation of surrogate data

To further validate that the proposed LSTM networks are in fact learning the underlying patterns in EEG sequences, the experiments were repeated on two types of surrogate datasets. First, random sequences of microstates were generated where each state was independently sampled with equal probability of occurrence. Second, for a more realistic imitation, a sequence of microstates was modeled as a discrete autoregressive process (Jacobs & Lewis, 1983), where the state at time *t* is a function of previous states and thus introduces correlations. In this case, the microstate sequence is assumed to be DAR(*p*) as $x_{n}=V_{n}x_{n-A_{n}}+\left(1-V_{n}\right)y_{n}$ where *x*_*n*_*ɛ* (*A*, *B*, *C*, *D*) is the *n*th state in the sequence, *V*_*n*_ is a Bernoulli process taking value of 1 with probability ρ and 0 with probability (1 − *ρ*). *A*_*n*_ is an integer between 1 and *p* attaining each value with probability *α*_*i*_, and *y*_*n*_ is another random process with independent and identically distributed probabilities of selecting a particular state, represented by marginal distribution π. The parameters of the autoregressive process are estimated from the original EEG microstate sequences by first mapping the symbolic states onto a set of numerical values.

## 3 RESULTS

### 3.1 Reconstruction of microstates sequences within each subject

We first analyzed the *Datasets 1A and 2* within an intrasubject framework where data from each subject was separately used for reconstruction. Four reconstruction models were trained for each subject with sequence lengths of 200, 400, 800, and 2000 ms with both intermediate and original representations. For intermediate representation, sequence length that best approximates the duration (200 ms, 400 ms, etc.) is considered as an exact match is not always possible due to the encoding process. To train a model for each subject, 70% of the data was used for training and the remaining were used as the test set. Then each set was further divided to overlapping segments of preset length and then randomly shuffled. We repeat this procedure for the intermediate representation as well. The performance of the models is measured using reconstruction accuracy which is defined as:

$$\text{Reconstruction Accuracy}=\frac{\text{number of correctly predicted microstates}}{\text{length of the sequence}\;\left(\#\text{of time points}\right)}$$

**Figure 4:**
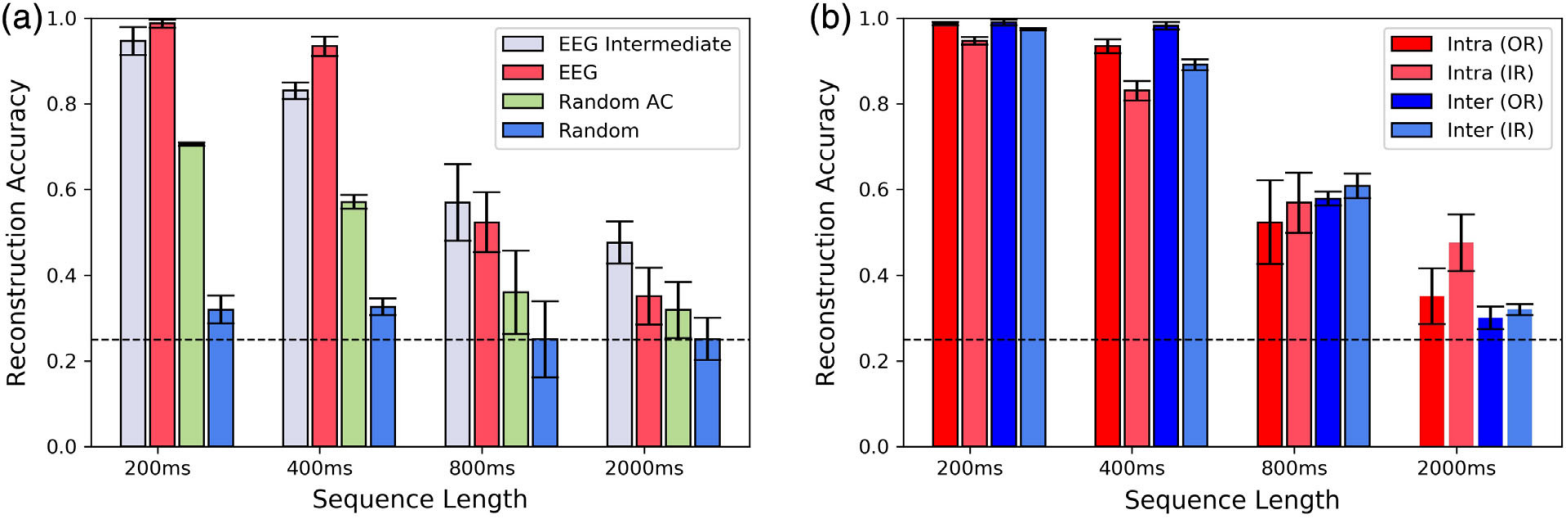
Electroencephalogram (EEG) original representation (OR) versus its intermediate representation (IR) versus surrogate data: For all different lengths of microstate sequences, the reconstruction (a) accuracies are significantly higher for EEG sequences as compared to random and random auto‐correlated (AC) sequences indicating the existence of strong underlying patterns. (b) Indicates intersubject and intrasubject reconstruction accuracy for different lengths of microstate sequences for original EEG and its intermediate representation. Consistently comparable reconstruction accuracies for intrasubject and intersubject analysis establishes the generalizability of patterns across subjects. Here, dotted line indicates the reconstruction accuracy of 25% for four microstates. For different time‐scales, EEG‐intermediate representation is in order with the original representation. This makes it clear that model does not only focus on persistence. These results further corroborate the existence of strong underlying patterns in EEG microstate sequences and demonstrate the ability of the LSTM‐based networks to effectively capture these patterns

The mean reconstruction accuracies across all subjects are depicted in Figure 4. Note that because within a microstate, periods between GFP peaks were labeled using a nearest neighbor criterion, within an average of about half the average GFP peak‐to‐peak interval, EEG time‐points receive the same microstate label by definition.

The reconstruction accuracy shows high stability for shorter sequence lengths of 200–400 ms and as expected starts to decline for 800 ms and drops significantly for sequence length of 2,000 ms. Our preliminary ablation studies (Appendix D) also indicate that varying the number units or layers of the LSTM network does not improve the performance of the model significantly for longer sequences of 2000 ms. Given that this decrease in accuracy is evident for both encoding schemes used here, this could not be attributed to the capacity of RNN. Here, to control for possible confounding effect of MRI artefact on the results of RNN microstate analysis, we compare the dynamics of microstates that are based on EEG recorded inside (*Dataset 1*) and outside (*Dataset 2*) scanner. We compare the accuracies of our RNN analysis between *Dataset 1* and *Dataset 2* (results presented in Appendix B) that are obtained from EEG data recorder inside and outside the MRI scanner and observe no significant difference. Additionally, we find no significant difference in the conventional metrics of microstate dynamics between the data recorded inside and outside the MRI scanner.

As the auto‐encoder attempts to learn stably occurring temporal patterns in the sequences, irregular bursts in the duration of microstates can affect its performance. As a result, we hypothesize that the rate of decrease in reconstruction accuracy can be attributed partly to the burstiness of the EEG sequences. Furthermore, empirical results show that for longer sequence lengths (2,000 ms), the accuracies for either intrasubject or intersubject reconstructions do not differ significantly from random auto‐correlated sequences. This observation suggests that memory effect in the microstate sequences decays rapidly beyond this duration.

### 3.2 Analysis of surrogate data

Reconstruction accuracy for random sequences is significantly lower than real EEG sequences (Figure 4). While the accuracy for completely random sequences (with equal probability of occurrence for each microstates) is close to chance that is, 25%, accuracies for autocorrelated random sequences is slightly higher.

### 3.3 Comparison of microstate sequences across subjects

Here, further analysis is done to test the extent to which microstate sequences are comparable across subjects. To do this, intersubject analysis is conducted where microstate sequences extracted from 80% of the subjects were used to train the RNNs and data from the remaining 20% of the subjects were used for testing the performance of the trained models with fivefold cross validation. Figure 4 shows the average reconstruction intersubject analysis and compares it with intrasubject analysis. Remarkable performance of the intersubject models indicates that microstate sequence trajectories are subject invariant at short time scales and can be generalized across subjects.

The higher reconstruction accuracy for intersubject as compared to intrasubject experiment can be attributed to the substantially greater number of training samples available to train the model. As microstate sequences from different subjects are pooled together, the large training sample enables the learning model to generalize better by capturing inherent variations more effectively.

### 3.4 Visualization and Interpretation of LSTM Cells

RNNs, composed of a large number of individual cells combined in complex ways to solve challenging tasks, are still majorly black boxes. With proliferation of large‐scale neural networks, interpreting them has become one of the most challenging and active areas of research (Karpathy, Johnson, & Fei‐Fei, 2015). LSTMs learn the underlying complex, nonlinear patterns by extracting high level features and generating rules directly from examples. Here, we attempt to gain some insight into the form of these rules by visualizing the hidden state representations of the network. Specifically, we tried to find LSTM cells that are dedicated or react to specific patterns in microstate trajectories.

**Figure 5:**
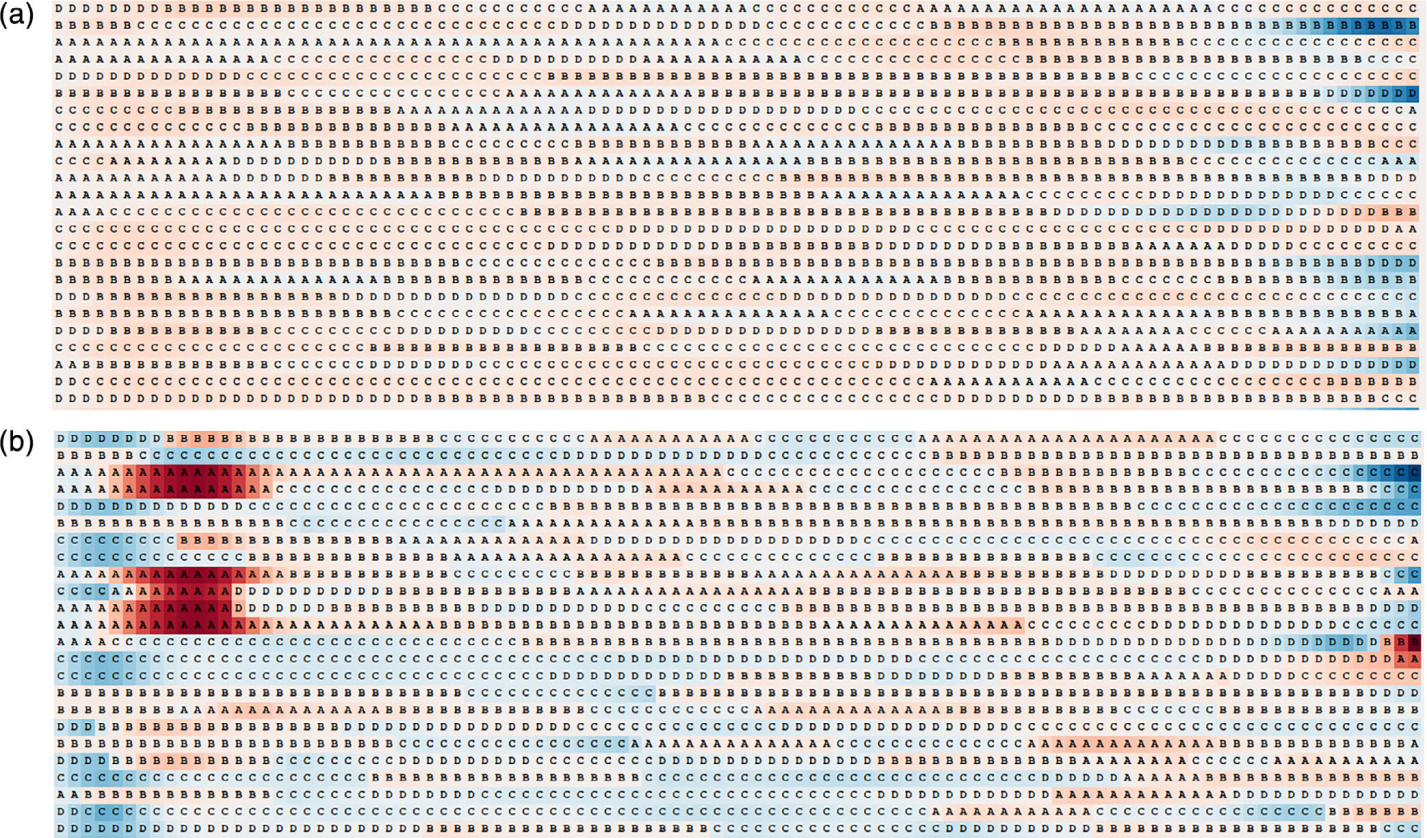
Visualization of two cells from hidden layer of the encoder. Each row represents a microstate sequence of length 100 where the number of examples represented by the number of rows is 32 and every microstate is highlighted by its corresponding activation value. The activation values of each microstate range between [−1, 1] and are represented by colors ranging from dark red (negative) to dark blue (positive). (a) This cell responds to the appearance of microstate A. (b) This cell responds to the transition to and from microstates C and D

Toward this end, the hidden layer of the network was visualized for better interpretation of the underlying lower dimensional representation of the temporal patterns. Specifically, we tried to visualize the internal units of a single layer, subject specific model for a sequence length of 400 ms only for the full EEG representation. As the LSTM reads a sequence of microstates, its cells fire with varying intensities. For each cell, these activations are visualized for an input sequence of microstates where each state is color‐coded according to the cell's intensity. These activation values range between [−1, 1] and colors range from dark red (negative) to dark blue (positive), respectively. Although many LSTM cells were too complex for visual interpretation, we were able to find multiple interpretable cells that robustly identified high‐level patterns. Remarkably, some of these patterns correlated with transition dynamics of EEG microstates. For example, Figure 5 depicts the activation of a cell that is sensitive to the occurrence of State A (Figure 5) and another cell that was found to track transitions to and from States C and D (Figure 5). For the purpose of visualization and ease of interpretation, these RNNs were trained with microstate sequences of length 100 in original representation.

For a more objective interpretation of these cells, we looked at how the activations were correlated with the temporal dynamics of the sequences. As a measure of state transition probability, average activation value for each transition (across a batch of sequences) for each cell *N* is calculated as.

$$S_{N:X\to Y\wedge \left(X\neq Y\right)}=\frac{1}{n}\left(\sum_{i=1}^{n}A_{\mathrm{Xi}}-A_{\mathrm{Yi}}\right)$$

where *A* represents the cell's activation or intensity and *n* is total number of transitions that occur from a state *X* to state *Y*. Average activation value for each state is also calculated as a measure of rate of occurrence of a particular microstate, that is

$$S_{N:X}=\frac{1}{n}\left(\sum_{i=1}^{n}A_{\mathrm{Xi}}\right).$$

**Figure 6:**
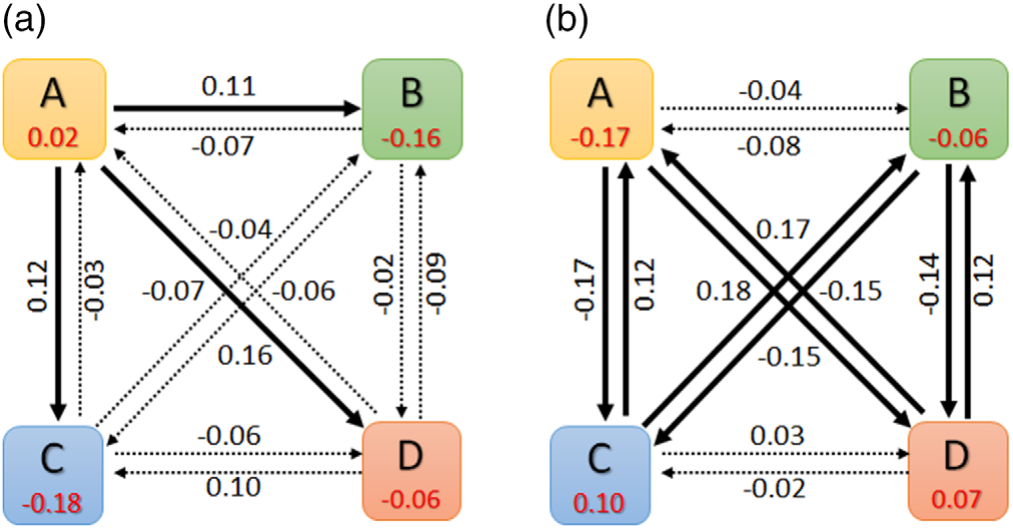
The block representation of average state activation values for each microstate and state transition matrix for hidden cell 1 (a) and 2 (b). Here, A, B, C, and D represent each microstate and values in the rectangular box represent average state activation values. The arrows from one state to another represent corresponding average state transition activation values. Arrows in bold indicate transitions that are different, that is, either too low or too high for a particular cell

Figure 6 depicts the activation metrics calculated for the two cells presented in Figure 5. The visualizations clearly show the responsiveness of LSTM units to multiple patterns. As can be seen from Figure 6, only State A has a positive average activation value with all transition activations from State A (A → B, A → C, A → D) , are positive and relatively large compared to other transitions, clearly indicating the interest of this cell in tracking the occurrence of State A. Similarly, the average activation values in Figure 6 indicate the specialization of that cell in tracking transitions to and from States A and B toward States C and D.

### 3.5 Effect of social stress on trajectories of microstates

Several studies have shown that temporal dynamics of the EEG microstate sequences are altered due to disturbances of mental processes associated with neurological and psychiatric conditions. Specifically, statistics such as duration and occurrence of microstates would be affected in such conditions. More recently, variations in transition probabilities between microstates have also been associated with aberrant neural dynamics. In this section, we use the previous RNN model to see if trajectories of the microstates are different in the resting state data following an exposure to a psychosocial stress task compared to baseline resting state. The stress response to the psychosocial stress is induced using the ScanSTRESS paradigm (Streit et al., 2014), which uses arithmetic as well as mental rotation tasks (see Section 2.1 for details).

**Figure 7:**
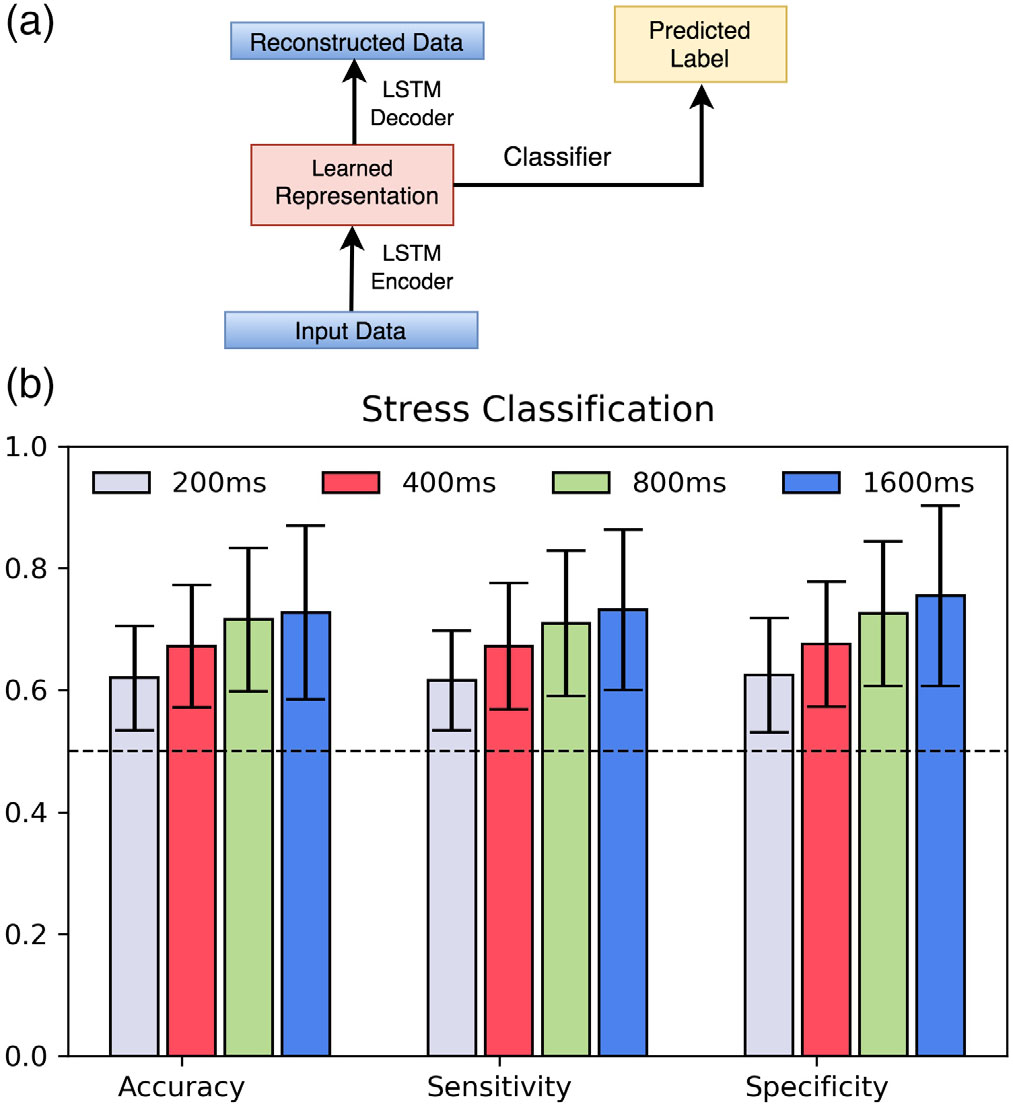
Block diagram of joint stress classification along with reconstruction. The reconstruction architecture is same as the one used for reconstruction but here we have coupled the system with pattern classifier which classifies the two conditions. Here, we back propagate both reconstruction and classification error at each epoch. The bar plot in the right indicates obtained accuracy, sensitivity, and specificity for classification at 200 to 1,600 ms. We observe that model is able to differentiate at all sequence lengths when compared to the simple statistical analysis where no difference was seen

Here, we hypothesize that since stress suppresses certain modes of activity in the brain (Olver, Pinney, Maruff, & Norman, 2015; Sandi, 2013; Yu, 2016), resting state data before stress condition (baseline resting state) should have a richer repertoire of microstate sequences compared to the resting state following the stress task. To test this hypothesis, we tested the generalizability of the RNNs to classify the sequences as stress or nonstress. As shown earlier (see Section 3.3), the time course of microstates seems to be comprised of multiple time scales where shorter time scales are subject‐invariant. Therefore, we repeated this experiment with four different sequence lengths of duration of 200, 400, 800, and 1,600 ms, respectively. We used an RNN Encoder‐Decoder coupled to a pattern classifier as shown in Figure 7. The combined model uses the latent representation of the AE to perform classification. This model is jointly trained in an end‐to‐end manner where both the reconstruction loss and classification loss are back‐propagated at each epoch. While the configuration of the auto‐encoder remains the same as that of the previous sections, the classifier is designed as a two‐layered dense neural network with leaky RELU as the activation unit and binary cross entropy as the loss function. The classification accuracies range from 67 to 73% for sequence lengths 200–1,600 ms, respectively.

**Figure 8:**
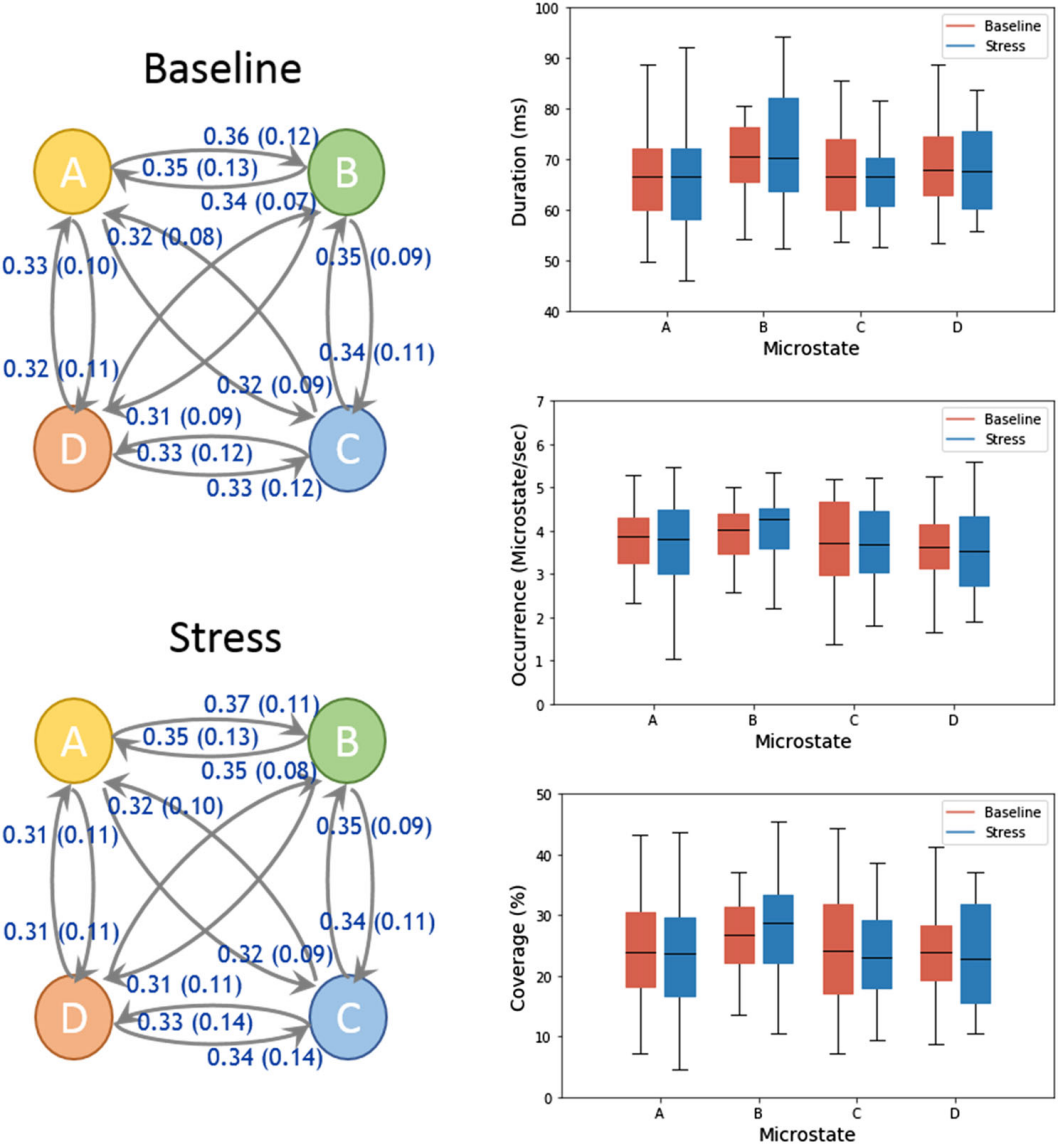
Transition probabilities, average duration, coverage, and occurrence frequency of microstates for baseline and stress conditions are compared. These results show that there are no statistically significant differences in any of the features between the two conditions

Consistent with the theory that functional brain states are suppressed under stress conditions, we observe that RNNs encodings obtained from joint training can distinguish between the EEG with or without preceding stress condition. Interestingly, the plot shows a clear trend of increasing accuracy with increasing sequence lengths and thus, emphasizes the importance of long‐range correlations in characterizing these sequences. Importantly, the effect of stress was not significant in any of the conventional measures (see Figure 8). Moreover, we attempted to classify stress versus nonstress for 1,600 ms sequences using simple neural network with conventional measures as input features. With architecture similar to that used for joint stress classification resulted in an accuracy of approximately 52% only.

To further illustrate the sensitivity of LSTMs to the trajectories of microstates, we conducted a cross‐conditional analysis. We tested the generalizability (Olver et al., 2015) of the RNNs when trained on these two datasets (with and without preceding stress) and tested on the other one. The summary of the results is shown in Appendix E. We observed that although when tested on the same session that the model was trained on, the two models have a similar performance (*p* < .43), the test accuracy of the model trained on data with preceding stress is significantly (*p* < .023) lower when tested on the session with no preceding stress. Interestingly, this drop in the accuracy is only observed when we cross tested the two models trained on the longer sequence length of 800 ms. These results suggest that RNNs trained on EEG data following the stress condition have less ability to generalize to data with no preceding stress and thus, further substantiating the idea of suppressed brain states under stress conditions.

## 4 DISCUSSION

Microstates denote the quasi‐stable topography of scalp‐EEG that remain constant for approximately 80 ms and are believed to be the building blocks of adaptive chain of neuro‐cognitive states. Therefore, the trajectories of microstates are expected to follow certain (yet unknown) patterns and hence be trackable. Previous studies on EEG microstate transitions has indeed shown that the matrix of transitions among microstates is nonrandom, asymmetric, and systematically affected in different neuropsychiatric disorders (Lehmann et al., 2005; Nishida et al., 2013). Here, we used RNNs to capture the dynamics of microstate transitions with high temporal resolution. We capitalized on the ability of RNNs to recover consistent patterns of EEG microstates and provided insights about the temporal characteristics of the microstate sequences at different sequence lengths. Extensive experiments demonstrated that RNNs can capture the underlying structure of microstate sequences with high accuracy.

We observed that microstate trajectories are largely subject‐invariant at short time scales (≤400 ms) and that the reconstruction accuracy of the model decreases gradually with increasing sequence lengths. In particular, our results suggest that for longer sequence lengths (2,000 ms), the accuracies for either intra or intersubject reconstructions do not differ significantly from random auto‐correlated sequences. Interestingly, this approximation is in line with both (Gschwind et al., 2015) and (von Wegner et al., 2016) that estimate the long‐range memory effects to last up to 1,000 ms in EEG microstate sequences, suggesting the existence of long‐range correlations with finite memory content. Moreover, the increasing trend in the classification accuracy of stress condition from 200 to 1,600 ms further emphasizes the importance of long‐range dependence in characterizing the condition‐specific features of these sequences. Collectively, these results suggest a multiscale temporal dynamics of microstate sequences where microstate sequences at shorter time scales are subject‐invariant and therefore possibly reflect mainly the primary sensory information processes rather than the high‐level cognitive processing which are likely to be coded with longer sequences. This observation is in line with recent findings relating appearance of each microstates to specific functional networks and that temporal characteristics of certain microstates can be manipulated by certain tasks (Britz et al., 2010; Britz, Diaz Hernandez, Ro, & Michel, 2014; Seitzman et al., 2017). Taken together, these results provide converging evidence for the complex relation between microstates functional relevance and their sequences further consolidating the proposal that microstates are the building blocks of sequences which manifest brain cognitive communications (Michel & Koenig, 2017).

The bursting behavior and long‐range temporal dynamics of EEG microstate sequences have been elaborately demonstrated in works such as (Gschwind et al., 2015). Our preliminary experiments confirmed that our datasets exhibit similar properties (Appendix F). While long‐range dependencies play an important role in effectively encoding a given sequence as part of both reconstruction and prediction, the task of a predictor is significantly more challenging as it needs to forecast future microstates based on the past information. We hypothesized that burstiness of the sequences increases the chances of incorrect prediction causing the predicted sequence to rapidly diverge from the original.

Towards this end, another variant of the reconstruction model was trained to investigate the possibility of predicting the future trajectory of microstates. Here, while the encoder RNN analyzes the pattern underlying the past microstate trajectory using LSTMs, the task of the decoder is modified to forecast the future states. The prediction model was trained using microstate sequence length of 100 (i.e., 400 ms) for each subject to predict the next 400 ms (for details see Appendix C). The results confirm our hypothesis and indicate that prediction accuracies are relatively low for forecast lengths beyond 40 ms. Interestingly, relevant LSTM literature (Jiang, Deng, Simeone, & Nallanathan, 2019) from the traffic domain (characterized by regular bursts) have demonstrated how the level of burstiness affects the forecasting accuracy. Consequently, given our success to reconstruct sequences with notably longer duration, it is reasonable to attribute the steep decline in prediction accuracy to the irregular nature of the bursts in EEG domain. In the current datasets, this average peak‐to‐peak interval was 56.3 ± 1.2 ms, such that we expected a “baseline” predictable sequence length of 28.1 ms. These results are in line with other studies arguing that resting state EEG microstates show nonstationary behavior which seems to be due to the irregular and “bursty” nature of the microstate durations (Gschwind et al., 2015).

While RNNs are extremely powerful in processing sequential data, interpretability of their internal structure and learned parameters is very limited. We attempted to gain some intuition into how the proposed LSTM‐based network learns to reconstruct EEG based microsequences by visualizing the hidden state representations of the network. We noticed some interpretable patterns that correlated with the transition dynamics of EEG microstate. However, large number of cells with visualizations too complicated for human‐interpretability suggest that most cells are involved in processing multiple patterns depending on the context. As the interpretability of LSTMs improves with advancements in the domain of deep learning, it would be interesting to test those techniques in the context of EEG microstate sequences in near future.

Importantly, we notice that the same algorithm presented in this article can be effectively applied to relate data from simultaneous EEG and fMRI. A vertically stacked LSTM architecture that helps create a hierarchical feature representation allows for better understanding of relations between electrophysiological and hemodynamic processes at multiple time scales. Due to the loop or chain‐like structure, the RNNs are inherently deep in time, a feature that helps the state of the network to summarize the historical information. Analogously, stacking multiple RNNs on top of each other can be interpreted as introducing depth in space. Essentially, this approach allows for the hidden state at each level of the network to operate at a different timescale. This mechanism can potentially be a powerful tool for analyzing simultaneously recorded EEG and fMRI data.

Furthermore, we note that because microstates are assigned based on the interpolation of the nearest GFP peaks, the effective length of the sequences are much smaller than the size of the full sequence. To address this issue, we replicated the results for both original full length sequences and an intermediate form that represents a tradeoff between the original and compact versions (see Figure 2). Interestingly, comparable results for both forms indicate the ability and flexibility of the LSTMs to effectively model the EEG sequences. Nevertheless, to assign the microstate labels, one could alternatively assign microstate labels on a single time point basis which fully ignores the GFP signal and can in principle change the absolute duration of the microstates. We find it quite interesting to test if a different assignment scheme would qualitatively change the results we presented here.

To conclude, we show that EEG microstates can be reconstructed by RNNs and their trajectories show a multilayer temporal structure. This suggests that the information encoded in microstates is far beyond the conventional univariate measures (e.g., see Section 3.5). Microstates trajectories can be reconstructed optimally for sequence length of 400 ms but the accuracy drops significantly for longer sequences. This observation together with the low predictability of microstates strongly favors the theory of bursting behavior of EEG microstates. Based on these results, we suggest that the temporal structure of microstates could be governed by multiscale mental processes where short‐term processes which seem to be subject‐invariant manifest the basic sensory processes, midterm sequences for mental states (e.g., stress response), and long‐term sequences are possibly coding for personality traits. Although it remains beyond the scope of this article, we find it an extremely rewarding endeavor to find the processes that belong to each of these time scales and their temporal properties.

## CONFLICT OF INTEREST

The authors declare no conflict of interest.

## Supporting information


**Figure S1** Structure of a basic long–or short‐term memory (LSTM) unit described in (Graves and Jaitly, 2014). LSTMs have a chain‐like structure, but the basic repeating unit has a special internal structure that supports long‐ or short‐term memory. Each unit is composed of a memory cell, which is responsible for “remembering” and a set of gates that regulate the flow of information through the chain. At a particular time step t, it takes current input sequence X_t_ and previous hidden state H_t − 1_. Each unit maintains two states, cell state C_t_ and hidden state H_t_ that transfer information to the next unit. Additionally, each unit consists of input gate I_t_, forget gate f_t_ and output gate O_t_ that are responsible for adding, removing and filtering relevant information to the cell state respectively. The gates are composed of either sigmoid (σ) or tanh activation functions to optionally let information through. The detailed process of carrying information and memory forward is done using recursive process (Graves and Jaitly, 2014). While X_t_ represents the microstate at a particular time point, all others are internal parameters that are learned during the training of the network.
Figure S2 Intra‐subject reconstruction (A) and prediction (B) accuracy for different lengths of microstate sequences. Graphs depict mean reconstruction and prediction accuracies across all subjects and error bars represent standard deviations. Dotted line indicates the chance prediction accuracy of 25% for four microstates. Differences between EEG data collected inside and outside the MRI scanner are statistically insignificant.
Figure S3 Inter‐subject microstate sequence prediction accuracies for time scales ranging from 4 ms to 400 ms. As hypothesized, the prediction accuracy gradually decreases and stabilizes at approximately 40% for sequence lengths greater than 150 ms. EEG versus its intermediate representation versus surrogate data: For all different lengths of microstate sequences, the prediction (A) accuracies are higher for EEG sequences as compared to random and random auto‐correlated (AC) sequences, indicating the existence of underlying patterns predictable to certain extent. (B) indicates inter‐subject prediction accuracy for different lengths of microstate sequences of EEG in original representation (OR) and its intermediate representation (IR). Here, dotted line indicates the chance prediction accuracy of 25% for four microstates.
Figure S4 Intra‐Subject reconstruction accuracies: (A) For different sequence lengths as a function of number of units in a single layered LSTM network (B) For sequence length of 2000 ms as a function of number of layers in the LSTM framework with different number of units in each layer. The trends above indicate that varying the number units or layers does not improve the performance of the model significantly for longer sequences of 2000 ms.
Figure S5 Microstate duration distributions: (A) The durations of consecutive occurrences of Microstate A depicts irregular bursts (B) Histogram of durations for all the four microstates shows heavy‐tailed distribution suggesting long‐range dependence.
**Table S1.** Reconstruction performance of (Recurrent Neural Networks) RNNS when trained and tested on different sessions with and without preceding stress condition. Reported p‐values are uncorrected. The experiment was repeated with two different sequence lengths of 100 and 200 which correspond to a sequence duration of 400 ms and 800 ms respectively.

## Data Availability

The full data of the study are currently not publicly available due to ongoing additional analyses, however,individual summary data concerning this manuscript may be available on reasonable request from the authors.

## APPENDIX A: STRUCTURE OF LSTM UNIT 1

See Figure S1. In all experiments in this article, the encoder and decoder RNNs both have only one single hidden layer with each comprising of 40 LSTM units. The AEs were trained with mean squared loss function (to minimize the mean square error between the decoder output and the input sequence) and Adam optimizer with a learning rate of 0.001. The models were regularized using dropout at the rate of 0.2 (retain 80%) that was chosen based on an independent validation set. The network hyperparameters, including the number of units and layers, were chosen by tuning the network using a coarse grid search. All models were trained using NVIDIA GeForce GTX 1080 and Python‐based Tensorflow package (Abadi et al., 2016). Training a model took roughly 1 hr per subject.

## APPENDIX B: INSIDE‐OUTSIDE SCANNER EFFECTS ON RECONSTRUCTION 1

See Figure S2.

## APPENDIX C: EEG MICROSTATE SEQUENCE PREDICTIONS 1

As in the reconstruction model, encoder RNN analyzes the pattern underlying the past microstate trajectory using LSTMs but here, the task of the decoder is modified to forecast the future states. The encoder passes the learned representation to the decoder which is used to initialize the state of decoder module for sequence prediction. After being initiated with a dummy input at the first step, the decoder recursively generates the output sequence *O*_1_, *O*_2_, ⋯, *O*_*T*′_ of desired length *T*′. Again, the decoder used in prediction is conditional in nature. At every step, the decoder feeds the output *O*_*t* − 1_ obtained in the previous step as the input for the current update. The motivation to use conditional decoding is two folds: first, it allows the decoder to learn multiple target sequence distributions (Srivastava, Mansimov, & Salakhudinov, 2015), which is a necessary condition since more than one target can exist in a given input sequence, and second, data has strong short‐range correlations which are best modeled by a conditional predictor. We trained this model using microstate sequence length of 100 (400 ms) for each subject to predict the next 400 ms (See Figure S3). For intermediate representation, as one time‐step prediction is not possible, we predicted for 1 and 5 timesteps corresponding to approximately 20 and 80 ms. Additionally, there is a gradual decrease in the prediction accuracy as the length of the predicted sequence increases but it remains stably above chance level (see Appendix B).

## APPENDIX D: ABLATION—PRELIMINARY RESULTS 1

The performance of an LSTM network depends on a number of parameters inherent to the architecture. Due to limited computational resources, exhaustive ablation studies could not be performed. However, preliminary sensitivity analysis (See Figure S4) was conducted by varying the number of hidden layers and number of units per layer. These findings suggest that lower reconstruction accuracies for longer sequence lengths is due to the inherent nature of the EEG sequences rather than a limitation of the LSTM network parameters.

## APPENDIX E: CROSS‐CONDITIONAL ANALYSIS 1

See Supporting Information Table S1.

## APPENDIX F: BURSTINESS AND LONG‐RANGE DEPENDENCE 1

See Figure S5.
