# Supplementary material for: Investigating the temporal dynamics of electroencephalogram (EEG) microstates using recurrent neural networks
Source: Hum Brain Mapp. 2020 Feb 24;41(9):2334–46. doi: 10.1002/hbm.24949 (PMC7267981; doi:10.1002/hbm.24949)
Supplement: Supplementary file 1 — Figure S1 Structure of a basic long–or short‐term memory (LSTM) unit described in (Graves and Jaitly, 2014). LSTMs have a chain‐like structure, but the basic repeating unit has a special internal structure that supports long‐ or short‐term memory. Each unit is composed of a memory cell, which is responsible for “remembering” and a set of gates that regulate the flow of information through the chain. At a particular time step t, it takes current input sequence Xt and previous hidden state Ht − 1. Each unit maintains two states, cell state Ct and hidden state Ht that transfer information to the next unit. Additionally, each unit consists of input gate It, forget gate ft and output gate Ot that are responsible for adding, removing and filtering relevant information to the cell state respectively. The gates are composed of either sigmoid (σ) or tanh activation functions to optionally let information through. The detailed process of carrying information and memory forward is done using recursive process (Graves and Jaitly, 2014). While Xt represents the microstate at a particular time point, all others are internal parameters that are learned during the training of the network. Figure S2 Intra‐subject reconstruction (A) and prediction (B) accuracy for different lengths of microstate sequences. Graphs depict mean reconstruction and prediction accuracies across all subjects and error bars represent standard deviations. Dotted line indicates the chance prediction accuracy of 25% for four microstates. Differences between EEG data collected inside and outside the MRI scanner are statistically insignificant. Figure S3 Inter‐subject microstate sequence prediction accuracies for time scales ranging from 4 ms to 400 ms. As hypothesized, the prediction accuracy gradually decreases and stabilizes at approximately 40% for sequence lengths greater than 150 ms. EEG versus its intermediate representation versus surrogate data: For all different lengths of microstate sequences, the pred [file HBM-41-2334-s001.docx]

# Appendix A: Structure of LSTM Unit

In all experiments in this paper, the encoder and decoder RNNs both have only one single hidden layer with each comprising of 40 LSTM units. The AEs were trained with mean squared loss function (to minimize the mean square error (MSE) between the decoder output and the input sequence) and Adam optimizer with a learning rate of 0.001. The models were regularized using dropout at the rate of 0.2 (retain 80%) that was chosen based on an independent validation set. The network hyper-parameters, including the number of units and layers, were chosen by tuning the network using a coarse grid search. All models were trained using NVIDIA GeForce GTX 1080 and Python based Tensorflow package (Abadi, et al., 2016). Training a model took roughly 1 hour per subject.


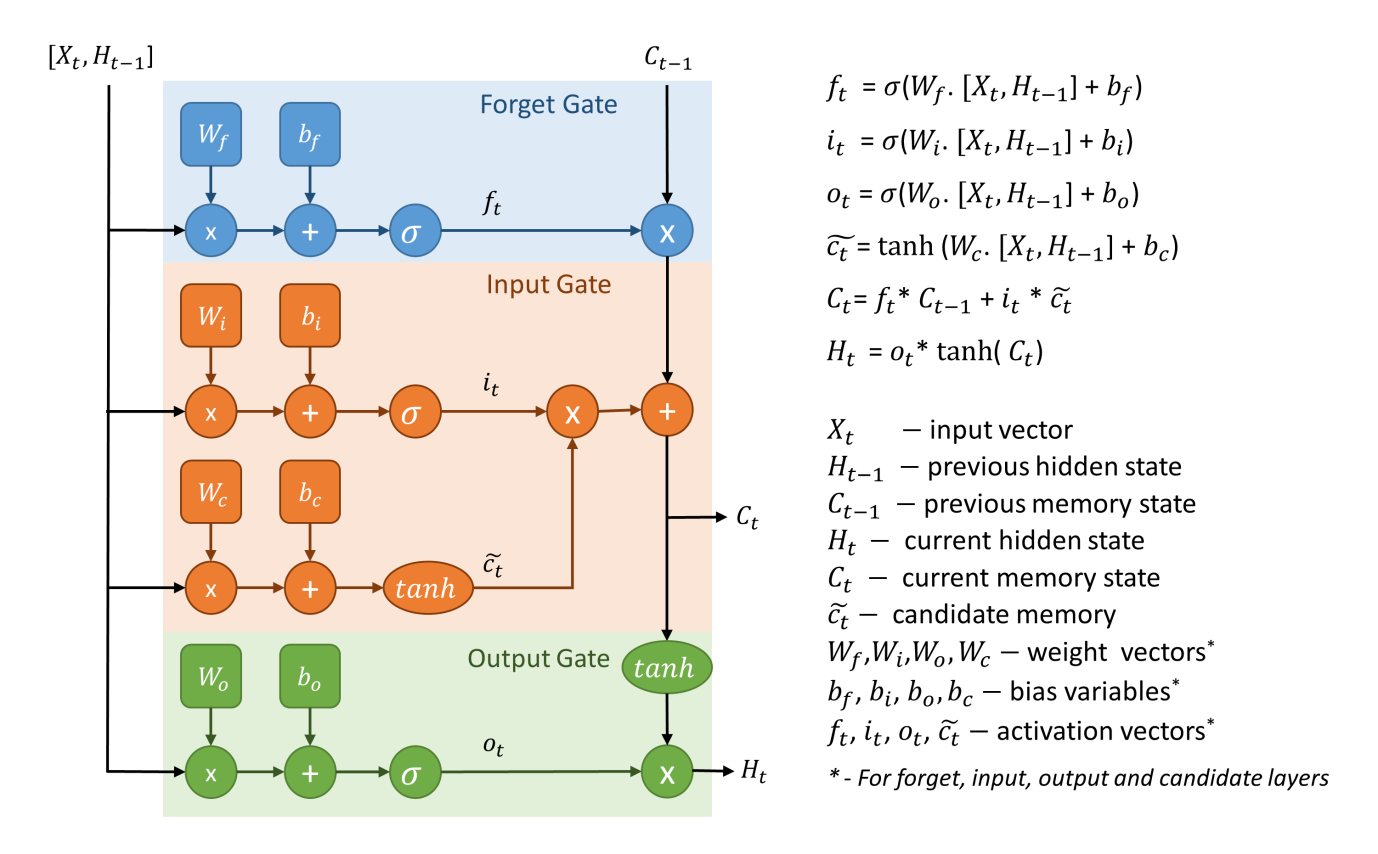


*Supplementary Figure 1: Structure of a basic long–or short-term memory (LSTM) unit described in (Graves and Jaitly, 2014). LSTMs have a chain-like structure, but the basic repeating unit has a special internal structure that supports long- or short-term memory. Each unit is composed of a memory cell, which is responsible for ‘remembering’ and a set of gates that regulate the flow of information through the chain. At a particular time step* $t$*, it takes current input sequence X_t_ and previous hidden state H_t−1_. Each unit maintains two states, cell state C_t_ and hidden state H_t_ that transfer information to the next unit. Additionally, each unit consists of input gate I_t_, forget gate f_t_ and output gate O_t_ that are responsible for adding, removing and filtering relevant information to the cell state respectively. The gates are composed of either sigmoid (σ) or tanh activation functions to optionally let information through. The detailed process of carrying information and memory forward is done using recursive process (Graves and Jaitly, 2014). While X_t_ represents the microstate at a particular time point, all others are internal parameters that are learned during the training of the network.*

# Appendix B: Inside-Outside Scanner Effects on Reconstruction


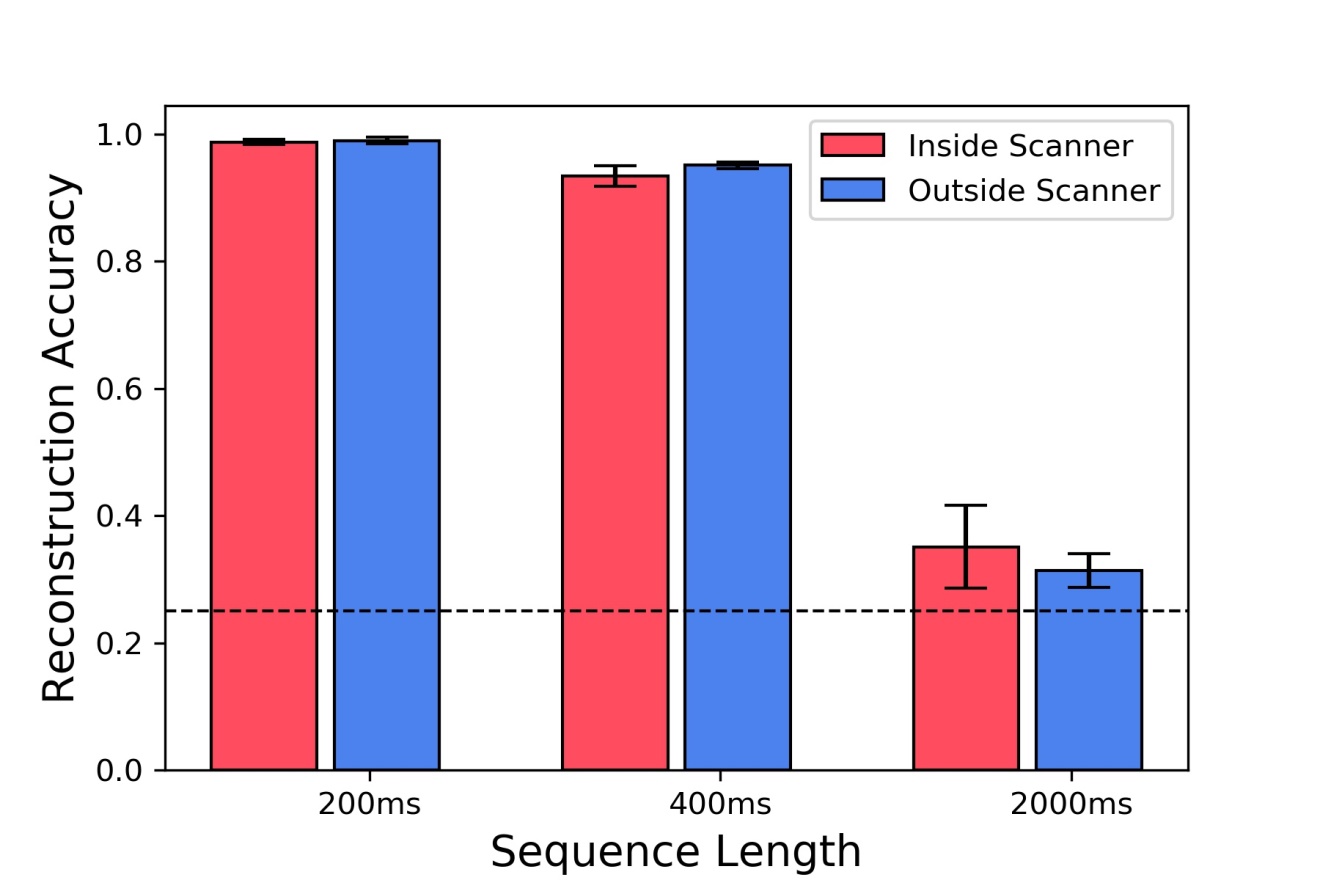

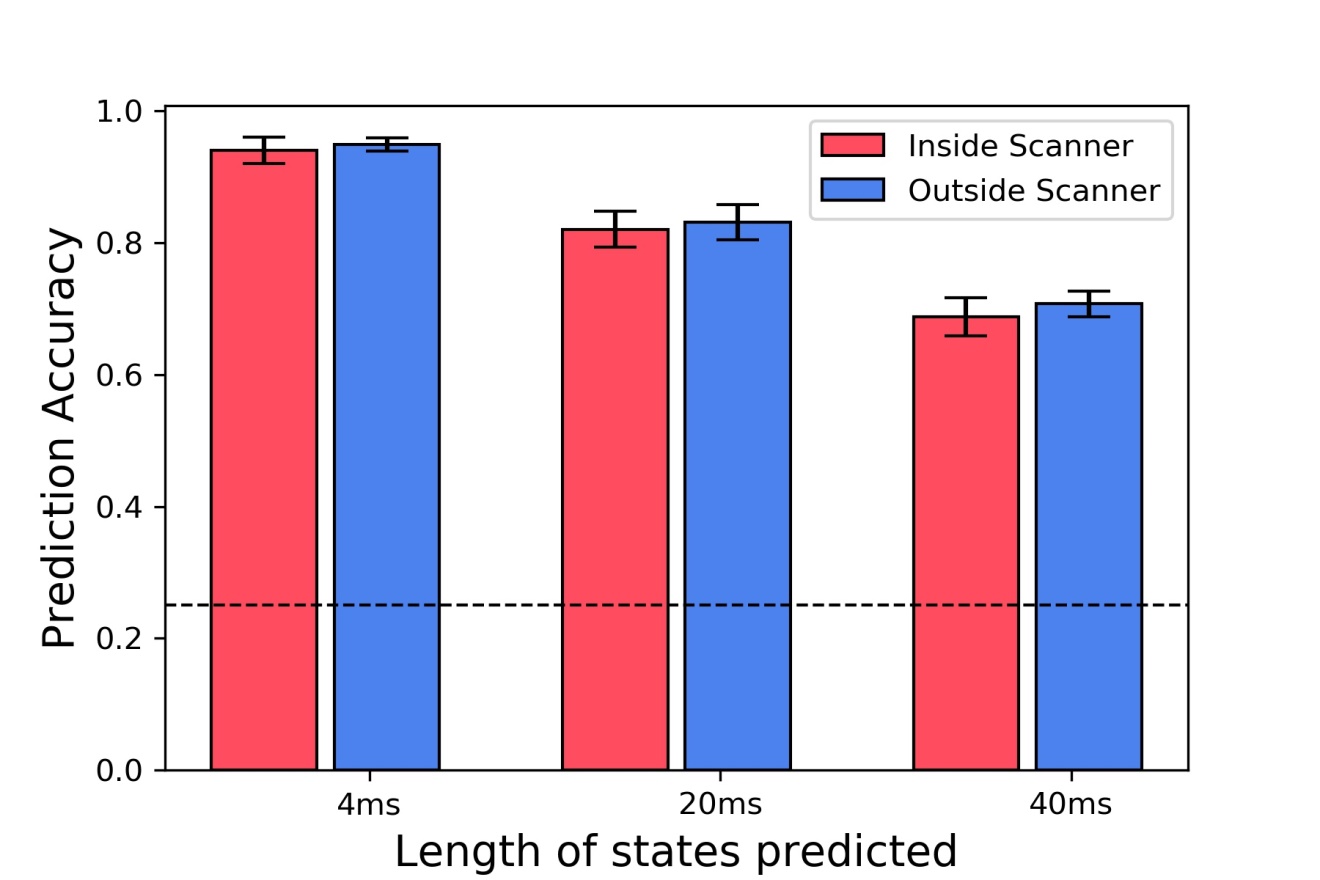


*Supplementary Figure 2: Intra-subject reconstruction (A) and prediction (B) accuracy for different lengths of microstate sequences. Graphs depict mean reconstruction and prediction accuracies across all subjects and error bars represent standard deviations. Dotted line indicates the chance prediction accuracy of 25% for four microstates. Differences between EEG data collected inside and outside the MRI scanner are statistically insignificant.*

# Appendix C: EEG microstate sequence predictions

As in the reconstruction model, encoder RNN analyzes the pattern underlying the past microstate trajectory using LSTMs but here, the task of the decoder is modified to forecast the future states. The encoder passes the learned representation to the decoder which is used to initialize the state of decoder module for sequence prediction. After being initiated with a dummy input at the first step, the decoder recursively generates the output sequence $O_{1},O_{2},\cdots,O_{T'}$ of desired length $T’$. Again, the decoder used in prediction is conditional in nature. At every step, the decoder feeds the output $O_{t-1}$ obtained in the previous step as the input for the current update. The motivation to use conditional decoding is two folds: first, it allows the decoder to learn multiple target sequence distributions (Srivastava, et al., 2015), which is a necessary condition since more than one target can exist in a given input sequence, and second, data has strong short-range correlations which are best modeled by a conditional predictor. We trained this model using microstate sequence length of 100 (400 ms) for each subject to predict the next 400ms. For intermediate representation, as one time-step prediction is not possible, we predicted for 1 and 5 timesteps corresponding to ~ 20 ms and 80 ms. Additionally, there is a gradual decrease in the prediction accuracy as the length of the predicted sequence increases but it remains stably above chance level (see Appendix B).

*
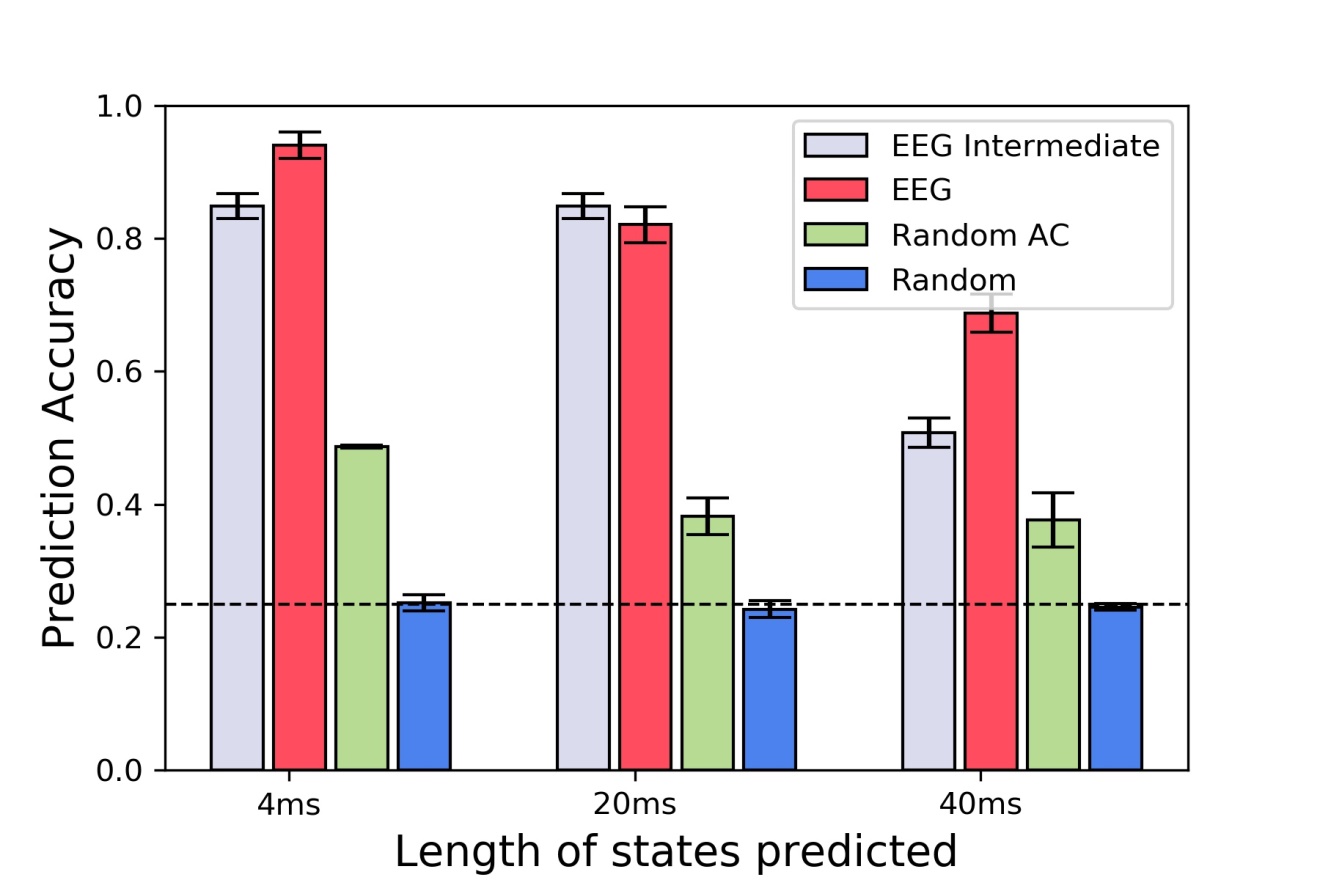

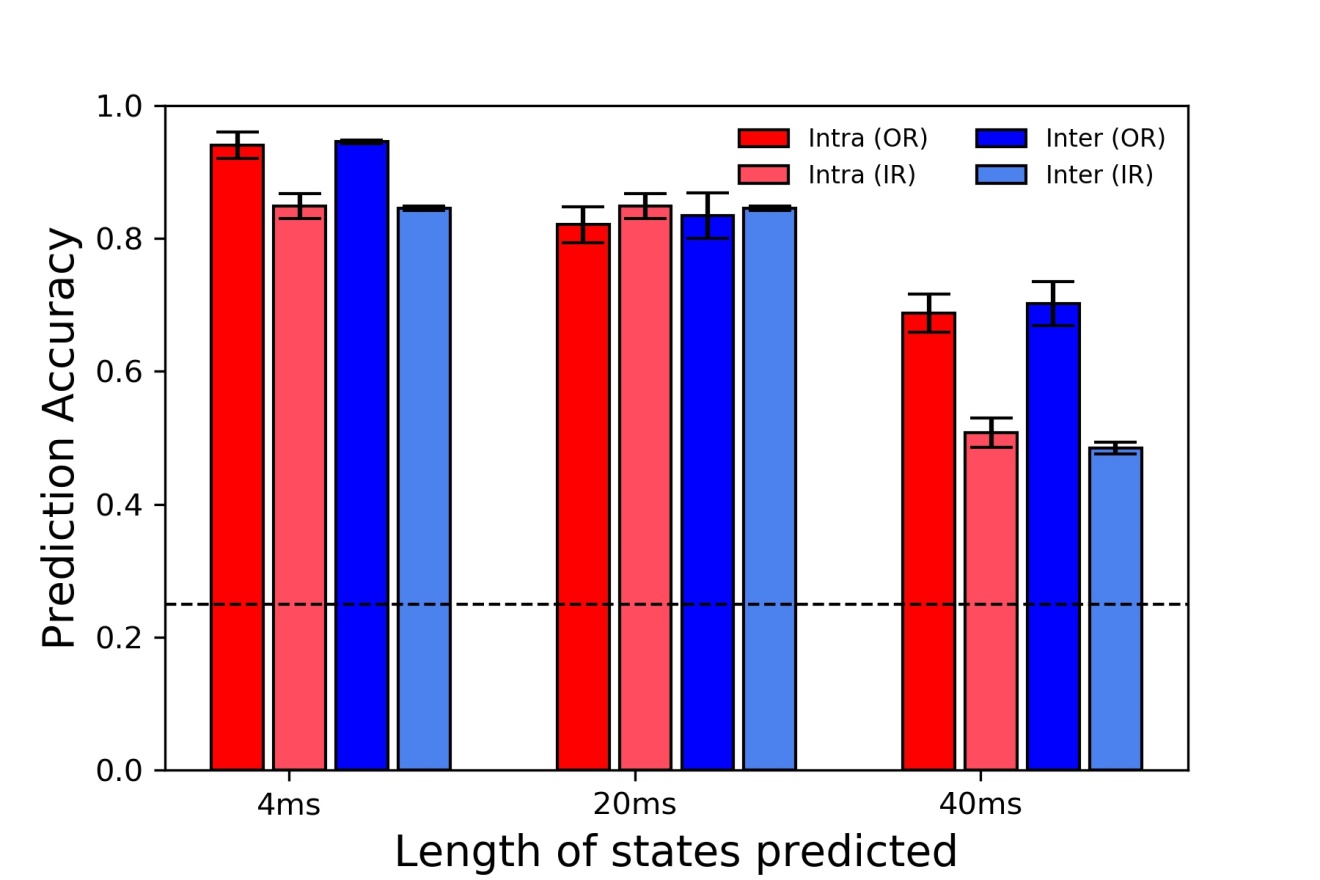

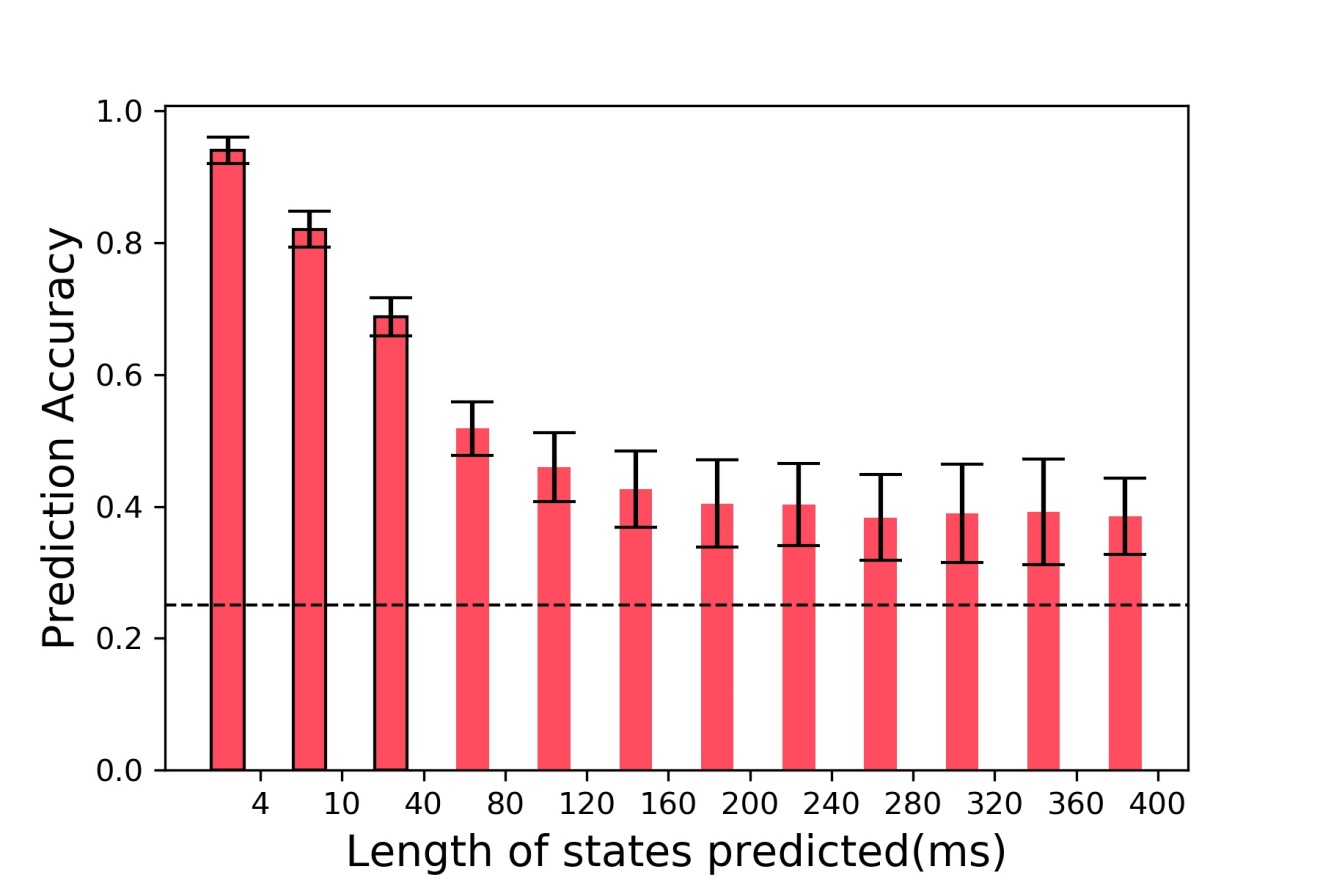
*

*Supplementary Figure 3: Inter-subject microstate sequence prediction accuracies for time scales ranging from 4ms to 400 ms. As hypothesized, the prediction accuracy gradually decreases and stabilizes at approximately 40% for sequence lengths greater than 150 ms. EEG versus its intermediate representation versus surrogate data: For all different lengths of microstate sequences, the prediction (A) accuracies are higher for EEG sequences as compared to random and random auto-correlated (AC) sequences, indicating the existence of underlying patterns predictable to certain extent. (B) indicates inter-subject prediction accuracy for different lengths of microstate sequences of EEG in original representation (OR) and its intermediate representation (IR). Here, dotted line indicates the chance prediction accuracy of 25% for four microstates.*

# Appendix D: Ablation – Preliminary Results

The performance of an LSTM network depends on a number of parameters inherent to the architecture. Due to limited computational resources, exhaustive ablation studies could not be performed. However, preliminary sensitivity analysis was conducted by varying the number of hidden layers and number of units per layer. These findings suggest that lower reconstruction accuracies for longer sequence lengths is due to the inherent nature of the EEG sequences rather than a limitation of the LSTM network parameters.

*
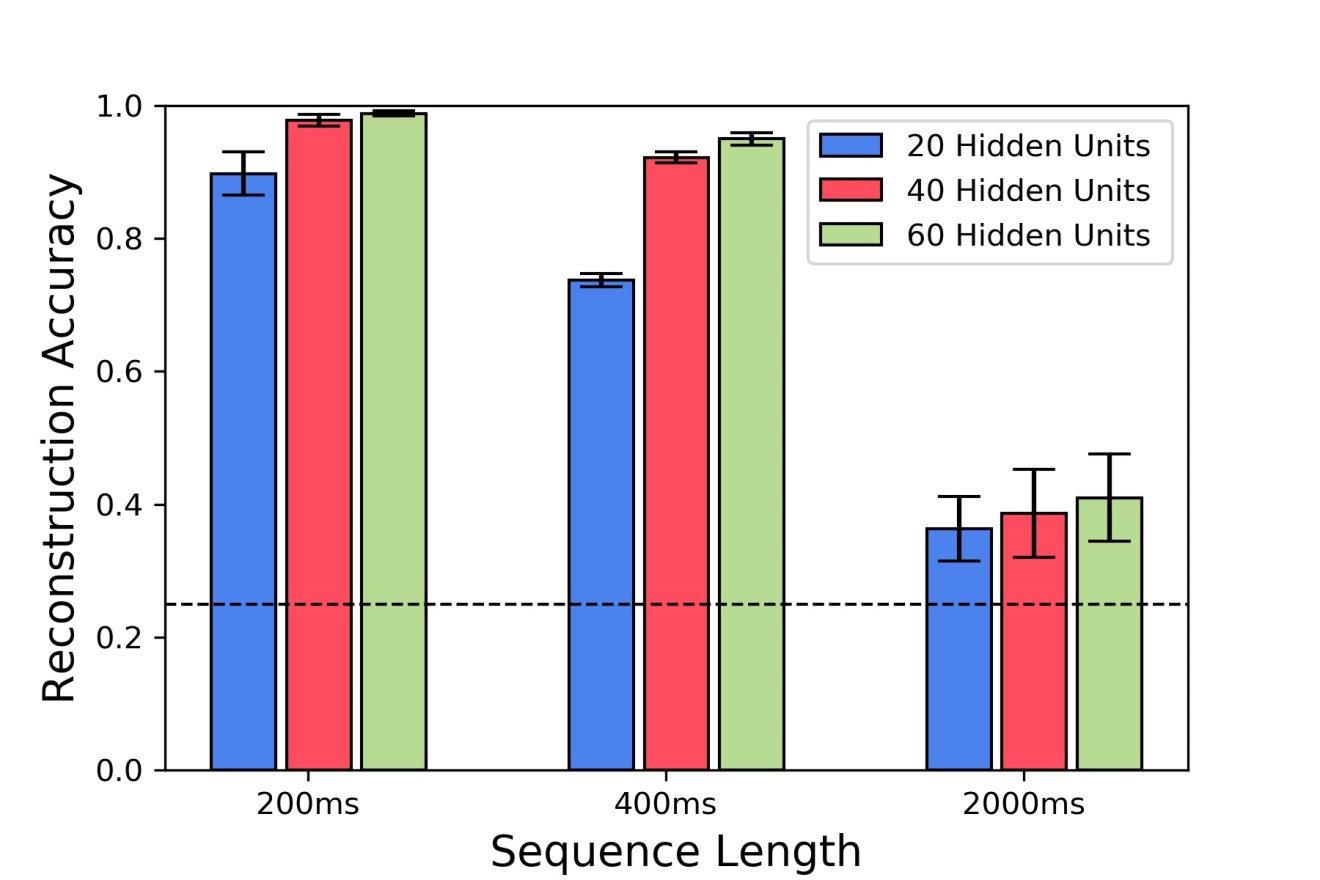

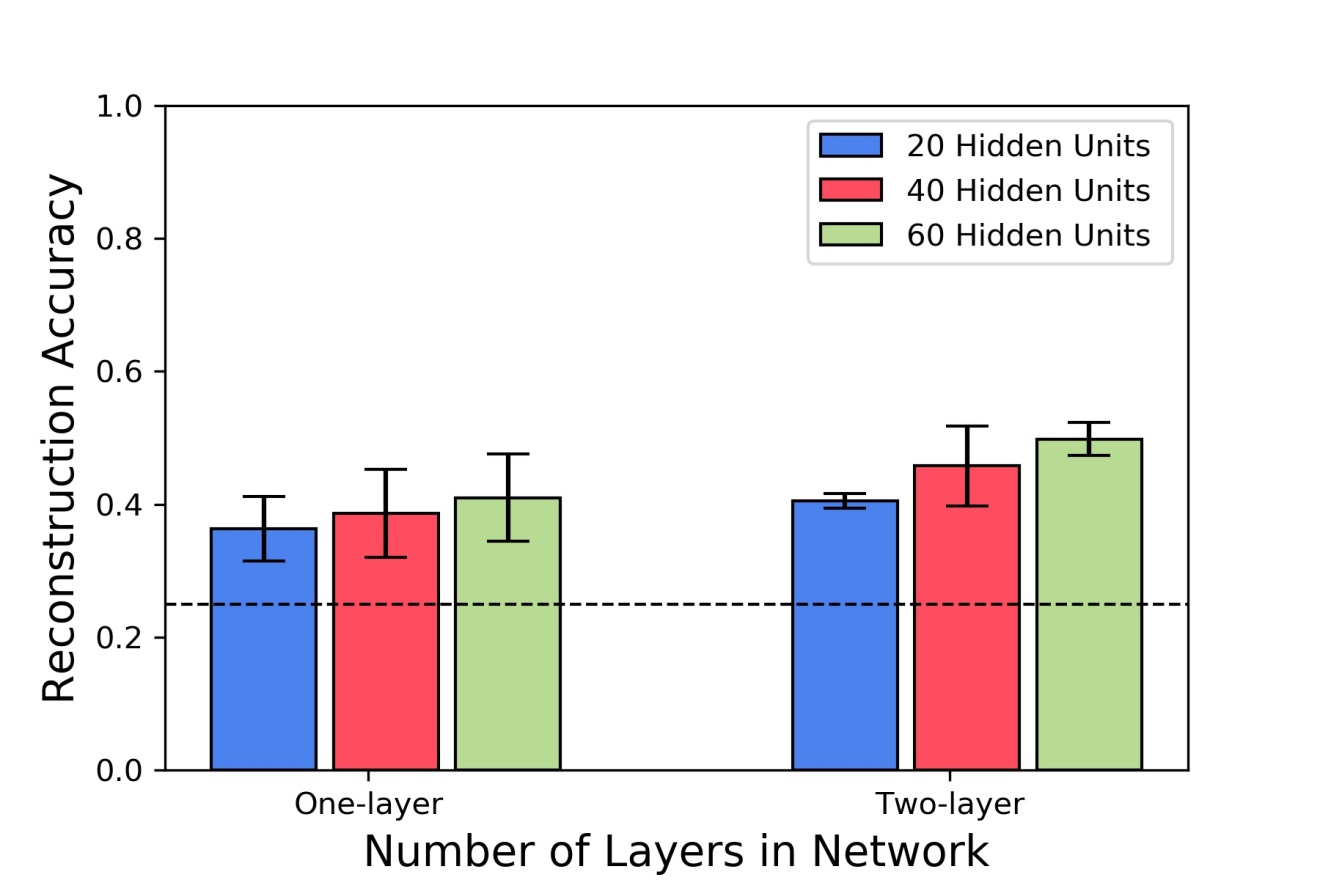
*

*Supplementary Figure 4: Intra-Subject reconstruction accuracies: (A) For different sequence lengths as a function of number of units in a single layered LSTM network (B) For sequence length of 2000 ms as a function of number of layers in the LSTM framework with different number of units in each layer. The trends above indicate that varying the number units or layers does not improve the performance of the model significantly for longer sequences of 2000 ms.*

# Appendix E: Cross-Conditional Analysis

*Supplementary Table 1: Reconstruction performance of (Recurrent Neural Networks) RNNS when trained and tested on different sessions with and without preceding stress condition. Reported p-values are uncorrected. The experiment was repeated with two different sequence lengths of 100 and 200 which correspond to a sequence duration of 400 ms and 800 ms respectively.*

# Appendix F: Burstiness & Long-range Dependence

*
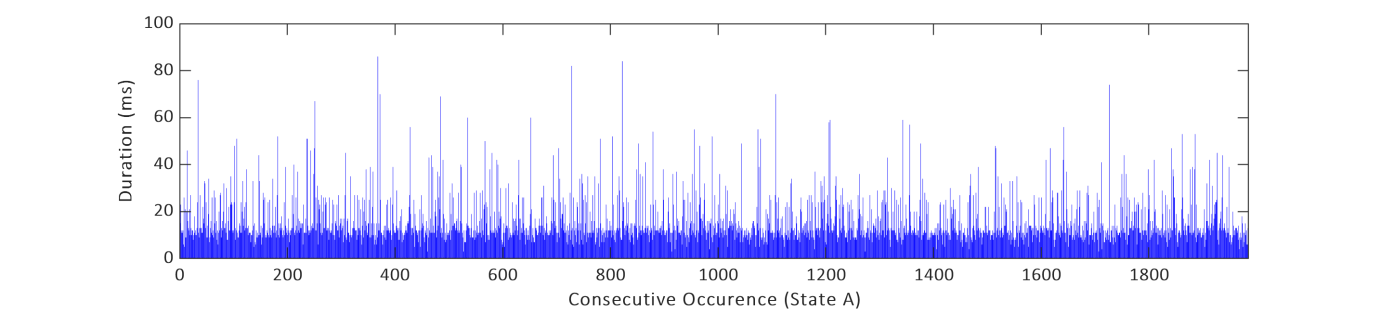

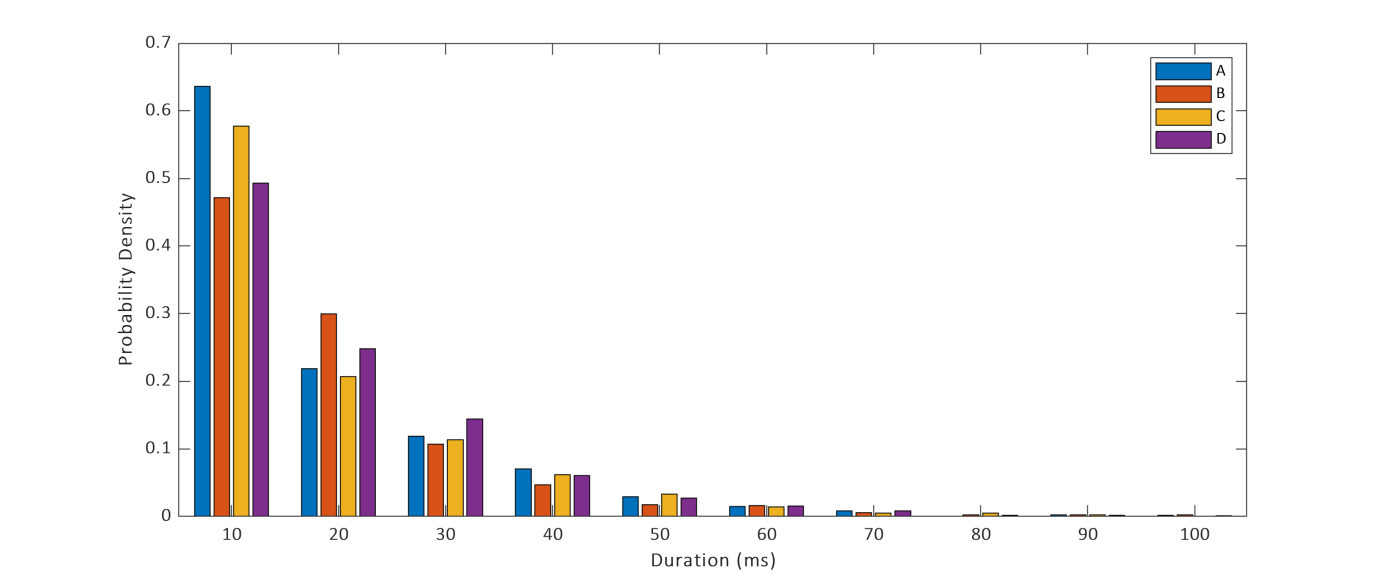
*

*Supplementary Figure 5: Microstate duration distributions: (A) The durations of consecutive occurrences of Microstate A depicts irregular bursts (B) Histogram of durations for all the four microstates shows heavy-tailed distribution suggesting long-range dependence.*
